# Book Reviews

**Published:** 1825-12

**Authors:** 


### CRITICAL ANALYSIS

OF

### ENGLISH AND FOREIGN LITERATURE,

RELATIVE TO THE VARIOUS BRANCHES OF

### Medical Science.

Quæ laudanda forent, et quæ culpanda, vicissim

Illa, prius, cretâ; mox hæc, carbone, *notamus*.—PERSIUS.

### DIVISION I.

### ENGLISH.

ART. I.—*An Essay on the Nature, Causes, and Treatment, of Water in the Brain.* By WILLIAM SHEARMAN, M.D. Member of the Royal College of Physicians, and senior Physician to the Royal West London Infirmary.—8vo. pp. 123. London: T. and G. Underwood, 1825.

NOTWITHSTANDING the frequency with which the disease denominated hydrocephalus has been discussed by different and able writers, the subject is by no means exhausted. There are yet many points of discrepancy to be reconciled, respecting both its nature and treatment. We have for many years been actively engaged in the treatment of infantile diseases, and have not been inattentive observers of the various maladies which have fallen under our care. Still not a single case of an acute specific disease, to which we could apply the oft-repeated term of hydrocephalus, has presented itself to our notice. As reviewers, we shall probably escape the charge of delivering singular opinions from a love of notoriety; for notoriety can only be sought by him who is known to the public, and can catch the murmur of approbation which any innovation upon received opinion is so likely to excite. We cannot venture to give any opinion as to what may have occurred in the practice of others. If we judge from the language of the day, or if we seek an opinion from recorded facts, we should infer that hydrocephalus, as an acute specific disease, is of almost hourly occurrence. If we judge from the evidence of our own senses,—from every circumstance that has fallen under notice, we should declare that we know not such a disease. We conceive it to be the imperative duty of every man to state, without reserve, the result of his own observation. We are not called upon to wrest the cases which we see to meet the distinctions which are laid down by nosological authorities. It has been stated by every writer upon the disease in question, that effusion of water within the brain is very frequently consequential upon many and different derangements of the general health of children: but still it is considered also as an acute specific disease, of no unfrequent occurrence. This is a part of the subject upon which we have long wished for more definite information, and it is with no ordinary gratification that we find it fully and fairly met by Dr. SHEARMAN, in the little but valuable volume before us.

In the Preface it is very truly stated, that water in the brain is either a more frequent occurrence, or more frequently suspected to exist, than was formerly the case. It might perhaps have been asserted, with equal propriety, that three-fourths of the cases which we hear of, and which adorn the records of even some of our public institutions, as "cases of hydrocephalus," are nothing more or less than cases of infantile remittent fever,—a disease which exhibits very frequently most of the symptoms that are said decidedly to indicate effusion within the cerebral cavities. It is the object of our author "to controvert the doctrine of water in the brain being a distinct specific disease, and to oppose the prevalent opinion of the proximate cause of the watery effusion being inflammation. It has been the author's endeavour to show that this symptom (water in the brain,) is an accidental occurrence, taking place in a great variety of diseases, and as the consequence of numerous causes acting upon the cerebral organs, depending upon a certain condition of those organs, constituting a state of predisposition merely, without the presence of actual cerebral disease." (*Preface*, p. vii.)

It is very true that "we are more occupied in vainly searching into the occult and abstruse causes of disease," than in watching with determined assiduity the rise, progress, and usual terminations of their symptoms, which is obviously so much better calculated to lead us to the great object of our wishes—a successful treatment.

To a certain extent, we are inclined to agree with Dr. Shearman in the following observations:—

"The author is aware that it will be considered almost heretical to express a single doubt of the superior value of anatomical pathology, compared with clinical observation, in obtaining an accurate knowledge of diseases; but thinking, as he does, that the latter is infinitely superior in this respect to the former, he is not deterred from avowing that opinion, notwithstanding its present unpopularity. He is not inclined to undervalue morbid anatomy as a means of adding to our knowledge, but he cannot go the length of elevating this branch of science to the important station which it occupies at present in the medical world, to the almost total exclusion of clinical observation, and neglect of the study of the natural history of diseases. Morbid anatomy is a useful handmaid, but she should not be injudiciously thrust up out of her station.

"That attempts have been made to do so, we have sufficient evidence in the zeal with which the superiority of morbid anatomy is advocated by some distinguished authors and lecturers; but it may be fairly put to these learned persons, whether observation and experience at the bedside be not surer guides to successful practice than morbid anatomy! To estimate their relative value fairly, let us suppose we were compelled to rely solely on one of these branches, which would prove of the greatest utility? It has been ascertained that a considerable progress may be made in knowing and curing diseases by the former source of information, independent of the other; but morbid anatomy *alone* would indeed be completely useless.

"An expert anatomical pathologist can, perhaps, tell us what will be the state of particular parts or organs, if any given disease be not cured: but does this knowledge, of itself, contribute any thing to our power of curing the disease? Are not the post-mortem appearances the same, or very nearly the same, when any appearances can be discovered, in all acute diseases, differing only in seat,—viz. redness, congestion, effusion, and suppuration, the effects of unconquered disease?

"We are no more assisted in our attempts to cure disease by the information that such will be the appearances, than by simply knowing that death will ensue if we be not successful in our endeavours." (*Preface*, p. xii.—xiv.)

But to proceed *ad punctum*, to lay before our readers an abstract of the opinions of Dr. Shearman upon the subject of hydrocephalus.

It is premised in the first chapter that an erroneous judgment of the actual condition of disease must unavoidably lead to injudicious practice, because our indications of cure can only be deduced from our established principles of pathology. If we confound with each other diseases, or states of diseases, essentially different in their nature and character, and look upon mere symptoms, or consequences of disease, as distinct and separate diseases, our treatment will be founded upon false principles.

The observations of Dr. Shearman are comprised under three general heads. The first contains remarks on the character and distinction of the malady; the second embraces an enumeration of some of its most frequent causes; the third briefly expounds the general principles of treatment, founded upon the views previously exhibited in the two former parts.

*Character and distinctions.*—In common medical language, the terms hydrencephalus and hydrocephalus, although they are etymologically different, are employed synonimously "to designate a series of symptoms preceding the actual effusion of water, and likewise those which are produced in consequence of the effusion. And hence arises the necessity of distinguishing the train of symptoms thus included under a term so comprehensively employed, into different stages, according to the successive order in which they appear.

"It would undoubtedly simplify our notions of the pathology of this affection, were we to confine the term hydrencephalus, or hydrocephalus, to its strict etymological signification, and employ it to designate merely the effusion, considering all the symptoms preceding actual effusion, however they may be produced, as the various causes of this effusion: to the removal of which causes our attention is to be directed, anticipating effusion as the final result of their continuance, should we be unsuccessful in our attempt to remove them." (P. 4, 5.)

Such a view of the subject is in accordance with the notions now entertained of the pathology of dropsy generally. It is considered as the result of disease, rather than in itself a distinct disease. "I am inclined to think," says Dr. Shearman, "that hydrencephalus, or water in the brain, is an accidental circumstance, occurring during the progress of several diseases, and is produced by a variety of causes; and that its occurrence in this case depends on the predisposition or previous state of the serous membranes of the brain in the individual; and that, therefore, the essential character of hydrencephalus, when the term is employed to designate any protracted series of symptoms, consists in that previous state of membranes, or predisposition." (P. 6, 7.)

It may be objected, and we think fairly, that the author enters too boldly into the field of speculation, when he ventures to state that "some previous state of the serous membranes" is the cause of effusion as a symptom of many other diseases. In truth, we know not why effusion rapidly succeeds a trifling derangement of health in one child; while in another, of apparently the same constitutional powers, no such circumstance occurs. It may depend upon relaxation of the exhalant arteries, or upon diminished absorption, or upon some alteration of the vital powers, as the consequence of venous congestion within the brain, or the suggestion of Dr. S. may be correct; but it is much better not to assume the appearance of knowledge, which in reality we do not possess. Granting only, then, that there is a predisposition to effusion within the brain, but not pretending dogmatically to lay down the essence of that predisposition, we fully agree with the following observations:—

"That the effusion is accidental, determined by the predisposition, not an essential result of the existing symptoms, is evident, because these symptoms frequently continue for a long period, and sometimes terminate fatally, without producing effusion, in those children who are not predisposed thereto by the peculiar state of the serous membranes, —viz. too great irritability and too augmented circulation.

"The different diseases, of which these various symptoms constitute a part, go through their natural progress, and terminate in their ordinary manner, when not influenced by the state of predisposition necessary to produce effusion. The worm or remittent fever of children has a regular progress, and appropriate terminations, of which effusion into the ventricles is not essentially one; yet, in children possessing that state of predisposition already hinted at, effusion is a frequent termination of this fever. In the latter case, the whole train of symptoms, including the fever, is usually called hydrocephalus; whereas, in reality, the fever was not any essential part of the hydrocephalic affection, but only an exciting cause.

"The same reasoning may be applied to various other diseases, in which water in the brain is found to occur or not to occur, according to the state of predisposition.

"The terms incipient hydrocephalus and confirmed hydrocephalus, and the division of the series of symptoms into different stages, including those which obviously take place before any fluid is effused, are clearly inconsistent with this plan of restricting the term itself to the actual effusion; but the extension of the term, so as to comprehend the very early symptoms of any disease which occasionally terminates in effusion, is also liable to many inconveniences, tending to confuse our ideas of the pathology of the affection, and consequently to lead to injudicious modes of treatment." (P. 8—11.)

We differ from the author as to the frequency of effusion as a termination of infantile remittent fever, provided that the disease is properly treated, and that the natural irritability, which exists to a great degree in every young child, is not excited to a fatal degree by the destructive exhibition of large doses of calomel, which are not unfrequently prescribed for the purpose of relieving a symptom which is yet to occur,—namely, effusion in the cerebral cavities.

"When the term hydrocephalus is not restricted to the actual aqueous effusion, but is employed so as to embrace all the various symptoms of preceding disease, as well as that state of predisposition which leads to effusion with facility, from slight causes, independent of preceding distinct disease, we possess no means by which we can form a diagnosis of hydrocephalus; and hence arise the contradictory statements we meet with, of the success experienced by different practitioners in their treatment of the supposed disease." (P. 12.)

The view taken by Mr. JOHN BURNS* of "secondary hydrocephalus," is essentially the same as that of Dr. Shearman.

All the symptoms which have been enumerated as constituting "acute hydrocephalus" have been frequently observed, and, upon examination of the patient after death, no fluid has been discovered. CHEYNE, QUIN, ROWLEY, WARREN, and GOLIS, have recorded many such cases. Our own practice would furnish, if it were necessary, similar examples.

"It is evident that, in these cases, the symptoms could, at no period of the disease, be produced by effused fluid; and we cannot, therefore,

---

* *Principles of Midwifery,* third edition, p. 547.

with strict philosophical deduction, conclude that in other cases, where effusion has been ultimately discovered, the symptoms, precisely similar in appearance and progress, were owing to the effusion. We have, indeed, strong grounds for supposing that the effusion was the accidental, not essential, termination of the disease; which, in all the cases, was probably *fever* of some description, affecting the brain in a greater degree than other parts,—a circumstance usual in that disease; in various forms of which, and in typhus among others, effusion sometimes takes place." (P. 16.)

As a proof that hydrocephalus is an accidental occurrence, we may appeal to the observations of the various writers upon that disease, who, when speaking of the treatment, observe that every form and every stage of the disease requires a considerable difference in our practice. In other words, the various derangements of children which may lead to effusion into the brain, require an appropriate variety in our treatment.

"Two conditions of brain may be considered as more particularly predisposing to effusion taking place during the progress of acute disorders in children,—viz. a greater rapidity of circulation in the vessels of the brain, and an excessive degree of irritability of the organ; and these states may exist either separately or combined, the danger of effusion being greater in the latter than in the former case. These states do not of themselves constitute disease, and may exist for a considerable time without any of the functions of life being impaired or interrupted, and are indeed compatible with the most perfect performance of such functions as are chiefly appropriate to the cerebral organ." (P. 26.)

Dr. Shearman occupies several pages in an endeavour to show that inflammation of the brain is rarely, if ever, followed by effusion. It is very true that inflammation is something more than an increased action of the vascular system; we must have resistance to the increased impetus. A contest then takes place between the resistance of the capillaries and the vis a tergo of the arteries, which precede them in the course of the circulation. We may easily conceive that the vis a tergo may not only be just sufficient to overcome the capillary resistance, and thus to terminate the inflammatory action by resolution, or a natural cure; but, in the struggle between these opposing powers, the vis a tergo may be so far increased as to impel the serous parts of the blood through the minute arterial extremities, and produce effusion, or hydrocephalus. We cannot doubt that inflammation of the brain frequently is followed by effusion. We are much indebted to Dr. Whitlock Nicholl for some very judicious remarks upon the "cerebral erethism" of infants. Where this condition of the brain exists, effusion is to be feared, from causes which might otherwise be applied without mischief.

The premonitory symptoms of an undue irritability of the brain, or increased circulation through its vessels, without the presence of actual disease, are thus enumerated by Dr. Shearman:—

"Great sensibility to slight impressions, with restlessness, and animation in a high degree; the retina of the eye is particularly sensible to light, and the pupil is sometimes much contracted; the child becomes fretful, and often cries without any apparent cause. In this state of brain, the organ readily manifests sympathetic affection with diseases of any part of the system: that is, slight causes of disease readily produce disturbance of the cerebral functions. Whenever fever arises from worms, dentition, costiveness, or any other cause, the brain participates in a disproportionate degree, and effusion is often the consequence; when, under a different state of the cerebral membrane, no such effect would have followed." (P. 52.)

The danger of effusion is rather in proportion to its rapidity, than to the quantity of fluid effused. When effusion takes place slowly, the brain may gradually accommodate itself to the pressure, and time is allowed for the removal of the fluid. "When the effusion is suddenly large, as in the water-stroke, the functions of the brain are at once speedily overwhelmed, and death ensues. Accordingly, the water-stroke is invariably fatal; the acute hydrocephalus may occasionally be cured: that is, in my view of the matter, the general disease of the system may be conquered previous to effusion coming on. In the water-stroke, the effusion taking place as the direct effect of the exciting cause, without any intermediate general disease of the system, and occurring with great rapidity, the case is rendered hopeless. Water-stroke is the actual original disease, not the accidental termination of some other disease." (P. 60.)

It is well known that, in protracted cases of chronic hydrocephalus, the firmness and elasticity of the brain are much diminished. In some instances, much of the cerebral substance has been removed, leaving little more than a mere membranous bag, or cyst, containing the effused fluid.

In the second division, the author enters upon the *causes* of water in the brain: they are divided into predisposing and occasional, or exciting; "and these latter may be further divided into such as act primarily on the brain, and into such as act primarily upon the general system, and remotely on the brain; or, as it is sometimes termed, by sympathy. Many of these causes are at once efficient, both in inducing the predisposition and exciting the actual effusion. In enumerating the causes, therefore, it will not be necessary to class them very strictly; but it will be sufficient to enter into a brief consideration of those circumstances which more frequently give origin, either as predisposing or exciting causes, to the affection in question.

"Fever, of whatever description, is one of the most frequent causes of effusion in the brain; and this result is not uncommonly discovered to have taken place, when we examine persons who have died of that disease. In children, particularly, we find hydrocephalus to result as a consequence of scarlet fever, a disease attended with a very considerable degree of vascular excitement, and in which the brain usually appears to be affected to a more than ordinary degree, as is evinced by the delirium which takes place even in comparatively mild cases of scarlatina.

"We do not find, however, that after an attack of small-pox or measles, when considerable hectic fever or chronic debility remains, there exists a greater disposition to hydrocephalus; it not being a frequent sequela to those diseases. This is owing, probably, to the state of contraction prevailing throughout the vascular system, and the exhalation being consequently diminished. In simple weakness, on the contrary, accompanied by universal relaxation of the vascular system, this affection is not an unfrequent occurrence.

"In children who are of very weakly constitutions, or of a scrofulous diathesis, where there exists a considerable relaxation of the lymphatic system, it is probable that, in consequence of the relaxed state of the exhalants, effusion may take place into the ventricles of the brain, disproportioned to the powers of absorption; and the accumulation may be so gradual as to allow the brain to accommodate itself to the increased pressure for a certain time, until mechanical resistance is offered to further distention; and then symptoms are produced, strictly those of hydrocephalus, inasmuch as they follow, not precede, the effusion.

"In proportion to the irritability of the system, is its liability to be affected by the exciting causes of diseases. Fever, therefore, more frequently exists in children, and is easily produced by very slight causes, as by fatigue, dentition, worms, &c. And hence arises a source of much error and ambiguity, because the fever thus produced, and of which effusion is sometimes the consequence, is mistaken for, and denominated the, early symptoms of hydrocephalus, by those who contend for its being a distinct and specific disease. But, as precisely the same symptoms frequently arise from the same causes, and are not attended with or followed by effusion, it is incumbent on those who discriminate between this train of symptoms and the specific disease (hydrocephalus), to offer some diagnostic signs whereby to distinguish the latter, previous to the arrival of the actual effusion, which has never yet been satisfactorily done." (P. 65—69.)

It may, in fact, be correctly stated, that whatever cause produces great general or local irritation, may be followed by effusion into the brain; the probability of this formidable symptom being, as we have before stated, infinitely greater in some children than in others.

Dr. Shearman throws out some very proper cautions against the excessive blood-letting, and free exhibition of calomel, which, in the treatment of infantile diseases, are so much the fashion with many practitioners, who act rather from opinions they carry to the bedside of their patient, than from those they form in consequence of their examination of the symptoms presented to them. Forced excitement of the mind in children is also very properly deprecated.

*General principles of treatment.*—The treatment of hydrocephalus must, of course, be regulated by the view which is taken of the essential nature of the disease.

"Those who believe the train of symptoms denominated hydrocephalus acutus to be a distinct specific disease, arising even in its earliest stages from inflammation, will resort to those depletory methods which are commonly employed to check inflammation; whilst others, who consider the effusion as an accidental circumstance, occurring in various diseases of different descriptions, and the symptoms preceding effusion as those belonging to such diseases respectively, and not constituting a distinct specific disease, (having effusion as its natural and essential termination,) will adapt their treatment to these particular diseases.

"The little success which has hitherto attended the attempt to prevent this ultimate and commonly fatal termination, by those who have treated hydrocephalus as a distinct specific disease, may incline us to bestow some attention upon the means naturally suggested by the consideration of the primary symptoms being those alone of some general or local disease, with which effusion has no natural or necessary connexion." (P. 95—97.)

If the train of symptoms has been preceded by worms in the intestines, or a costive state of those viscera, purgatives will be necessary. A combination of scammony, jalap, and calomel is preferred.

"But it is chiefly when costiveness and worms act as causes of fever, and produce this latter affection in the system, that doubt and ambiguity exist; the fever thus produced being mistaken for a specific disease, depending on inflammation of the brain, measures are sometimes employed which, however well adapted for the removal of inflammation, did it actually exist, are most inappropriate to the cure of fever; and we cannot, therefore, wonder at the frequent failure we witness of the treatment of hydrocephalic predisposition.

"Fever is a disease which, when once produced, goes on independently of its exciting cause, and has a certain natural progress and determinate duration. It consists of repeated exacerbations and remissions, and is naturally disposed to terminate on certain days, in preference to other days; and, in proportion to the regularity of its progress, is the chance of its earlier cessation. It is commonly terminated by what is called a crisis, which is more or less perfect according to the degree of artificial interference; and the danger of relapse corresponds to the more or less perfect state of its crisis. During the progress of the fever, various parts or organs are affected in a disproportionate degree compared with other parts or organs, depending on some peculiar state of such parts, obvious or concealed. Hence arises the necessity of employing measures to relieve the organs thus inordinately affected from threatened danger, although such measures may have no effect in shortening the progress, or perfecting the crisis, of the fever.

"When there exists in a child that predisposition in the brain which has already been mentioned, fever, once produced, affects that organ in an inordinate degree, similarly to what happens in adults, where we find sometimes the brain, sometimes the liver, sometimes other organs, exhibiting the *effects* of the fever; and the symptoms of such local affection, combined with those of the general fever, constitute the state denominated hydrocephalus acutus.

"It is under these circumstances that a diversity of opinion exists as to the relation this general and local affection bear to each other, as cause and effect. While some maintain the local affection to be the original disease, calling it inflammation of the brain, of which the febrile affection are the mere symptoms; it may, on the other hand, be contended that the local affection arises during the progress of the fever, and is a mere effect of the disease, determined in an inordinate degree to the cerebral organ, in consequence of its previous disposition." (P. 99—103.)

We are of opinion Dr. Shearman fairly urges that "Dr. Cheyne, notwithstanding his hypothesis of the nature and proximate cause of hydrocephalus, bears witness to the utility of remedies calculated to effect the intentions above mentioned; and he thereby undoubtedly confirms the view I have taken of hydrocephalus being a concomitant or sequence of proper fever. He says that antimonials, in combination with cathartics, have appeared to him very useful in the infantile remittent, and other cases *liable to degenerate* into hydrocephalus. This implies hydrocephalus not to be a distinct specific disease *ab origine*, but a termination of other acknowledged diseases, or an accidental circumstance arising during their course." (P. 105.)

Dr. Shearman confesses (in common, we presume, with every other physician,) that "whatever has a tendency to diminish the determination of blood to the cerebral organ, or to allay its excessive vascular action, must be highly beneficial in cases of threatened attacks of hydrocephalus." But he "strongly objects to general blood-letting in children, because this evacuation produces immediate weakness, and consequent increase of irritability." As a confirmation of the impropriety of general bleeding, Dr. Seeds' experiments are referred to, by which it has been proved that, on bleeding healthy animals to death, "a very large quantity of water was effused into the ventricles." We do not doubt the fact: it is what we should have expected *a priori* from the experiment. The exclusive objection, however, of Dr. Shearman to general blood-letting in children, appears to us to be expressed with singular want of caution, and to imply nothing more than that, if we bleed too largely, we shall do harm. Again, we differ from him in his opinion, that a moderate loss of blood, by means of leeches, will protect the brain from threatened danger, where there are evident signs of increased determination. He does not, indeed, trust to leeches alone for the cure of the malady. We have seen leeches applied in a very considerable number of cases, from the opposition of the parents to other modes of blood-letting, or the prejudice of the practitioner; and we are decidedly of opinion that no case of "evident determination of blood to the head" ought to be entrusted to their application. Either blood should be drawn by cupping or from the jugular vein, according to the age and strength of the child.

Dr. S. is of opinion that "no class of medicines appears to be better adapted to the combined state of fever and irritability than sedatives and diuretics; and considerable benefit has been derived from employing them in hydrocephalus. The mineral acids, united with squill or with digitalis, exert a beneficial effect both in diminishing the force of the exacerbation and controling inordinate vascular action, both general and local." (P. 114.)

Dr. Shearman's work merits the attention of the profession much more than many bulky tomes which are sometimes inflicted upon the public. The view he has taken of his subject appears to us, upon the whole, correct. There can be no doubt that hydrocephalus is, in a great majority of instances, a mere symptom of previous derangement or disease; but we do not feel ourselves justified in asserting that cases of idiopathic effusion *never* occur, because we have ourselves not witnessed them. We must be allowed, however, to depend so far upon the result of nearly twenty years' experience, as to deny altogether that acute idiopathic hydrocephalus is of such common occurrence as the statements of many practitioners would lead us to believe.

---

Art. II.—*Medico-Chirurgical Transactions.* Vol. XIII. Part I.— 8vo. pp. 280; with five Plates. Longman and Co. London, 1825.

[Concluded from page 423.]

In concluding our account of Dr. Marshall Hall's paper on the Effects of Loss of Blood in our last Number, we stated that the remaining contents of this volume consisted of little more than cases, "such as usually are, and ought to be, published in Medical Journals, rather than in the Transactions of learned Societies." This expression we have reason to believe has been regarded as implying rather a contemptuous opinion of the papers left unnoticed. Now, this was not our intention; although, on re-perusing the paragraph, we admit that it fairly admits of such an interpretation. A moment's reflection will convince our readers that *we*, of all men, would be the last to hold up the pages of our Journal as containing matter of inferior quality; and we confidently refer to the Original Communications which have appeared in this work, as contradictory of such an assumption. True it is that on the first of every month each subscriber must have a new Number on his table; and consequently the same selection cannot be observed as in those cases where only once a year, or more, a volume makes its appearance, containing papers culled by the united wisdom of the "council" from a whole harvest of communications. But we meant—and with this explanation we repeat the sentiment,—that cases of interest find a more rapid circulation by appearing in one of the periodical Journals, than when kept up till a sufficient Number has accumulated to make a decent-sized volume; while Essays and general views of science correspond better with the character which we naturally look for in the Transactions of a learned body. It was our intention to have given some of the remaining papers in our COLLECTANEA; but, as this department is preoccupied, we have thought it better to subjoin an analytic account of them.

13. ***History of a Case of Hydrometra and Dry Gangrene occurring in the same Individual.*** By Dr. A. T. THOMSON.

The following account is given of this patient:—

"She was a widow, her husband having died insane in 1819; up to which period she was of temperate and industrious habits, and enjoyed good health; but, on the death of her husband, she became intemperate in the use of spirituous liquors, and a martyr to rheumatism. She had married early in life, and it is important to know that she had borne two children. The symptoms under which she laboured, at the time of her admission into the infirmary, were those of acute rheumatism, affecting chiefly the knees and ankles. The degree of fever was considerable; and, being a woman of sanguine temperament, she was confined to bed, freely bled and purged, and kept on low diet. She took as medicine the colchicum, both in substance and in its vinous preparation, and occasionally the oil of turpentine; under which plan of treatment she was so much relieved in two months, that she was discharged convalescent. She did not, however, recover her strength; and, having probably returned to her former habits of intemperance, her health became again so much impaired, that five months afterwards—viz. early in December, 1823,—she was re-admitted into the infirmary of the workhouse.

"She appeared now somewhat emaciated; and complained of uneasiness and pain, connected with a tumor in the abdomen, which she informed Mr. Gaskell she had first perceived about six weeks prior to her admission into the infirmary in April, although, from a sense of delicacy, she had not mentioned it at that time. It was situate in the lower part of the abdominal cavity, rising, as it were, out of the pelvis, and occupying the iliac, hypogastric, and umbilical regions. She appeared as large as if six months gone with child. An indistinct fluctuation was perceptible in the tumor, and the least pressure on it excited pain. It was suspected to be a diseased ovarium; but no examination was made per vaginam; nor could it be ascertained, from the account which the patient gave of its origin, whether it had at first appeared on either side of the abdomen. The accompanying symptoms, however, denoted a greater derangement of the system than usually attends dropsy of the ovarium. These were—want of appetite, considerable nausea, furred tongue, the pulse quick and feeble, the bowels irregular, and the urine scanty and high-coloured. It was deemed expedient that she should be again confined to bed, and put on a vegetable diet. Mercurials were prescribed, so as to affect the mouth slightly; the bowels were kept open; and leeches and blisters were alternately applied on the abdomen over the tumor. By these means its increase was arrested; but the sensation of pain on pressure remained unabated.

"Matters proceeded in this manner until the middle of January, 1824, when the left foot was discovered to be affected with dry gangrene, which gradually extended nearly to the knee. I was now requested to see the patient. I found her in the state which has been described. The diseased foot and leg were much shrunk, and appeared more like parts of a mummy than of a living being; or as if a dark brown or blackish leather stocking were drawn over the limb of a skeleton; the whole being thin, dry, and harsh to the touch, and the natural divisions between the toes nearly obliterated. Although the state of the woman's health was not favourable for amputation, yet, as a feeble but distinct pulsation could be felt in the popliteal artery, and a line of separation between the diseased and the sound parts of the leg was fully formed, I concurred in opinion with Mr. Gaskell that the removal of the limb afforded the only chance of preserving her life. The operation was delayed, however, in consequence of the reluctance of the patient to submit to it; but it was at length performed on the 29th of February, four inches above the knee. Very little blood was lost, and every thing appeared to be doing well until the third day, when the strength of the patient suddenly sunk, and she died on the evening of that day.

"Before proceeding to detail the appearances on dissection, it may be proper to describe the state of the amputated limb. Its external aspect has been described. On cutting through the integuments, the parts beneath, cellular matter, muscles, ligaments, and blood-vessels, seemed changed into one undistinguishable ash-coloured mass, not unlike the internal texture of some of the dry vegetable fungi. In several parts, however, this was mixed with a semifluid matter. I endeavoured to trace the arteries, by injecting them from the sound part of the limb; but the line of separation was so complete as to render that attempt abortive.

"*Dissection.*—The first object which presented itself, on the abdominal parietes being divided and turned aside, was a body closely resembling the gravid uterus, occupying the whole of the pelvic cavity, and the greater part of the abdominal. Upon its anterior surface, and firmly adhering to it, was the urinary bladder, containing a small quantity of deep-coloured urine. On laying the flaps of the abdominal parietes together, the stretched bladder was found to extend within an inch of the umbilicus; so that it must have been perforated, if the trochar had been employed to evacuate the fluid during the life of the patient, under the supposition that the disease was ovarian dropsy. The tumor was immediately ascertained to be the uterus, greatly enlarged and filled with fluid. It was partially sphacelated in its peritoneal covering, on the upper portion or fundus.

"With regard to the other viscera, the liver was much diminished in size, and adhered to the diaphragm throughout; the gall-bladder was large, and turgid with deep-coloured bile; the stomach, colon, and other intestines, with the omentum, were glued together in many places, and in some were in an evident state of sphacelation. This gangrenous appearance extended to the peritoneum, in the hypochondriac region. On removing the diseased uterus from the body, and making an incision into it, the quantity of fluid which it contained was found to measure eight quarts. It was of a dark brown colour, and coagulated slightly when heated in a spoon over the flame of a candle. The existence of a large hydatid within the cyst was suspected; but this opinion was incorrect, the sac being merely the uterus, in the cavity of which the fluid was contained. The internal surface of the organ was not more irregular nor more spongy than in its natural state; but none of the orifices could be found, for even the os uteri was interiorly as *completely obliterated as if it had never existed;* and, although its situation could be traced in the vagina, yet even there it was very faintly marked. The ovaria were small and flaccid, but otherwise natural.

"On examining the state of the larger blood-vessels within the abdomen, in reference to the diseased limb, the aorta was discovered to be ossified about two inches above its division into the iliac arteries. The right iliacs were in a healthy state; but the left external iliac was ossified in several places, in some of which the bony portion embraced nearly the cylinder of the vessel. The ossific matter appeared to be deposited between the innermost and the second coats of the vessels. No traces of ossification were observed in the course of the femoral artery, and, as has been already stated, this vessel pulsated in the ham, although the lateral vessels, the muscular and articular arteries, which are given off from the popliteal artery, were shrunk and nearly obliterated." (P. 171—176.)

The case is followed by some interesting pathological remarks, and references to other analogous affections.

14. *Cases of Destructive Inflammation of the Eye, &c. occurring in the Puerperal State.* By Dr. Marshall Hall and Mr. Higginbottom.

These gentlemen have witnessed together the progress of several cases of ophthalmia, with suppuration of the integuments, occurring from five to eleven days after delivery. In every instance, the inflammation was preceded by serious constitutional derangement: in one case there were intestinal irritation and uterine hemorrhage; in another, there was long-continued diarrhœa; and in others, fever, with derangement of the bowels. One patient had been bled; another used calomel freely; a third, opium; and in a fourth the oleum terebinthinæ had been administered. In all, the left eye was the one affected; and it is suggested as a possible cause of this, that the patients had all been delivered in the usual position,—viz. lying on the left side. We confess that we do not see how this could have had any influence. In one, the eye was inflamed only for a day or two before the patient's death; in two, there was chemosis to a great extent, the eye appearing collapsed during life; in another case, the patient survived ulceration and sloughing of the cornea, the entire destruction of the eye, and the subsequent healing of the anterior part.

A very remarkable feature in this formidable disease consisted in a local inflammation of the integuments, first observed on the hand, but soon afterwards found on the inferior as well as superior extremities. In one case only there was no such cutaneous inflammation; yet even there an orifice of a vein, which had been opened for the purpose of blood-letting, inflamed and suppurated.

All the remedies tried were unavailing; among which were early bleeding, mercury, purgatives, and afterwards bark and opium.

From these observations it is conjectured that the disease originated in constitutional causes, particularly as all the patients had previously suffered from some indisposition. Five cases are detailed, which present considerable variety in their history: we select the first, as giving a general idea of the malady.

"Mrs. A., aged twenty-three, of a delicate constitution, was delivered, after a natural labour, on the 27th of October, 1823. Before the time of her confinement, she was affected with continued diarrhœa, for which she took occasionally a dose of rhubarb. On the ninth day after delivery, an affection of the left eye was first observed: red vessels were seen proceeding across the conjunctiva, and converging towards the cornea; the pupils were extremely contracted, and there was great intolerance of light.

"On the succeeding day, the course of the enlarged vessels was completely obscured by great tumefaction of the tunica conjunctiva; the transparency of the cornea was somewhat lost, the pupil enlarged; the pulse was very frequent.

"On the third day of this disease, the chemosis, or tumefaction of the conjunctiva, was greatly augmented. Large red vessels were observed passing in all directions; the cornea was still more opaque, uneven on its surface, covered with a film of mucus, sunk in the chemosis, and surrounded by a ring of ulceration about a line in breadth, and covered with white pus. The pupil was still more enlarged, but seen obscurely through the opaque cornea; there was still intolerance of light, and the vision was very imperfect. The eyelids were greatly swollen, partly perhaps from the application of leeches; the pulse 144.

"On the fourth day, the eye was nearly in the same state; the pulse was only 120, and the patient expressed herself as feeling much better.

"On the morning of the fifth day, the pulse was 100; in the evening, 120. During the night there had been slight delirium: the chemosis was less, the ulceration round the cornea greater, the cornea more opaque, uneven in surface, and irregular in form; the pupil could not be distinguished. A little pus was observed in the lowest part of the anterior chamber; there was less appearance of mucus over the eye.

"On examining the eye in the gentlest manner on the sixth day, the cornea gave way, and the aqueous humour and crystalline lens escaped, and flowed down the cheek. The chemosis now subsided, and the remaining portion of the cornea collapsed.

"In a day or two afterwards, chemosis increased, and there occurred great pain, redness, and tumefaction, and complete closure of the eyelids. This again subsided, and, when the eyelids could be separated, the eyeball appeared of a brownish hue, the space previously occupied by the cornea presenting the appearance of a white ulcer. In the course of a week or two, the swelling totally subsided, the conjunctiva became white, and the ulcer healed.

"About the second day of this affection of the eye, the wrist and arms became affected in five distinct points, by a diffused redness and great tenderness. In the course of a few days, suppuration took place, and each part required to be opened by the lancet. Afterwards most copious suppurations took place in the integuments in different parts of the surface of the body, along the thigh and legs, in the axilla, and above the clavicle. These suppurations eventually exhausted the patient, and she died in a state of extreme feebleness and emaciation.

"This patient took opium for the diarrhœa; was bled and took mercury in the beginning of the affection of the eye; and afterwards had cinchona and wine." (P. 192—195.)

15. *Case of Carotid Aneurism, successfully treated by tying the Artery above the Aneurismal Tumor.* By Mr. WARDROP.

This is one of the boldest operations to be found in the records of modern surgery, and the case is unquestionably very interesting and important. It is remarked by the author, that in those cases where it is impossible to reach the artery between the heart and the aneurismal tumor, (instances of which are by no means very rare,) it has been customary to leave the patient to the horrors of his fate. But, says Mr. Wardrop,

"As in those where the aneurism has been cured, either spontaneously or by tying the artery between the tumor and the heart, the curative process has been effected by the coagulation of the blood contained in the tumor, it is remarkable that surgeons should not have taken advantage of the knowledge of this fact, and, in those cases where the artery could not be tied between the tumor and the heart, have tied it beyond the tumor.

"If we suppose a case of femoral aneurism in the middle of the thigh, it is easy to conceive that, when the ligature of the artery at a distance from the tumor was first contemplated, it might have been a matter of dispute whether the ligature ought to be applied below or above the aneurismal tumor; for it is just as easy to imagine that the blood contained in the space between a ligature placed below an aneurismal tumor and the first arterial ramifications above the tumor, should coagulate, as that the blood contained in the space between a ligature placed above the tumor and the first anastomosing branch below it, should undergo the process of coagulation. Besides, there are even advantages that might have been anticipated by tying the artery beyond the tumor, in such a case. In the first place, the danger of secondary hemorrhage at the place of tying the ligature would be less, from the little resistance it would, at that part of the vessel, be required to make to the impulse of the blood, in comparison to what would be necessary were the ligature placed between the tumor and the heart. In the second place, every collateral branch between the tumor and the heart would be saved to carry on the future circulation; branches which must be obliterated were the artery tied at some distance above the tumor." (P. 217—219.)

The result of the case has fully proved the legitimacy of this reasoning; and, as it is the first successful operation of the kind which has been placed on record, we should consider it a great omission were it not to be found in the pages of our Journal.

"A lady, seventy-five years of age, after a very violent fit of coughing, perceived a swelling on the right side of her neck, a little above the clavicle. When I saw her, eight days afterwards, the tumor had all the characters of an aneurism of the carotid artery, and had become as large as a fist; but was so situated that it was quite impracticable to tie the vessel below the tumor, so closely did it come in contact with the clavicle. The tumor continued to increase in size, and, on the eleventh day after it was first observed, it had acquired a formidable aspect; the scapular portion having become very red and painful, the pulsation, which was very strong throughout the whole swelling, being here particularly so, and the parietes feeling extremely thin, and as if ready to burst.

"It was evident that the patient's life was now in the most imminent danger; and, in this hopeless condition, it forcibly struck me that it might be highly expedient to tie the carotid artery above the aneurism, in the hope that, by thus stemming the current of blood through the vessel, nature might establish a new channel to carry on the circulation, allow the blood in the tumor to coagulate, and the sac and the vessel to contract and be obliterated, as take place after the common operation. There were circumstances which made this case particularly favourable for resorting to such a measure: the aneurism had been of short duration,—the patient, though far advanced in years, had a healthy constitution,—she was of a tranquil disposition, and eager that something should be done for her relief. Besides, the diseased artery was most favourable for the proposed operation; for, as no branches are sent off from the carotid artery until it divides into the external and internal, the process of coagulation would not be interrupted by the continuance of circulation through collateral branches in immediate contiguity with the aneurism. The operation also appeared practicable in this instance, as the aneurism, though large, extended upwards so as still to leave sufficient space for the application of a ligature between the tumor and the division of the artery.

"Under these impressions, and with the approbation of Dr. Veitch and Mr. Glen, who also attended the patient, I undertook the operation; and the result of it has, in my opinion, fully authorised the measure, and I trust that the future experience of others will confirm its utility.

"The operation consisted in making an incision through the skin and cellular membrane, rather more than an inch and a half in length, commencing it immediately above the tumor, and extending it on the tracheal edge of the mastoid muscle, and in the direction of the carotid artery, taking care to avoid the large superficial veins. The subsequent part of the dissection was chiefly made with a silver knife, guided by the finger; and there was no particular difficulty in reaching the artery but what might have been anticipated, from its great depth, from the necessary limits of the incision, and from the numerous large veins which were carefully to be avoided,—particularly a branch which extended across the middle of the incision to the internal jugular, and which consequently diminished the space in which the artery was to be taken up. After a careful dissection, which was tedious from its being necessary to tear the parts with the silver knife, the artery was so completely separated from the adjacent parts, that the point of the finger could be readily passed between the vessel and the vertebræ, and the aneurismal needle was passed round the artery with singular facility, taking care to avoid the par vagum, which was distinctly felt behind the finger. The vessel being previously ascertained to be healthy, one ligature was tied round it, as close to the tumor as the incision would admit, and the lips of the wound were stitched together by a suture, without any farther dressings. The aneurismal tumor was covered with adhesive plaster, in order to protect the tender skin, and at the same time to keep up a certain degree of pressure.

"I thought it probable that the resistance to the circulation, which the ligature would necessarily occasion, might, for a short while at least after its application, be followed by an increase in the distention of the tumor; instead of which, however, there was an immediate decrease in its bulk, marked by a considerable corrugation of the skin at the base, as well as a diminution of its redness. The ligature of the artery did not seem to produce any change in the mental functions, or any unnatural feelings in the head: on the contrary, the patient passed the night after the operation more comfortably than that previous to it, the tumor being accompanied with less uneasiness.

"A progressive diminution in the bulk of the aneurism, and in the strength of its pulsations, took place, so that, on the fourth day after the operation, it seemed to have diminished nearly one-third in its bulk; the upper and tracheal portions had lost all pulsation, and only the scapular portion retained an obscure undulatory thrill. The integuments, which had lost their redness, now evidently became more inflamed; and, during the fifth and sixth days, there was a distinct increase in the size of the tumor, and it pulsated more strongly, which seemed partly owing to several severe fits of coughing. This apparently unfavourable change was, however, followed by a decided amendment; and, eight days after the operation, the swelling again began to diminish, and the pulsation became more obscure, so that on the fourteenth day it was not much larger than half its bulk at the time of the operation, and no pulsation could be detected in any portion of it; merely a slight vibration in some parts, which seemed to be produced by the pulsations of the contiguous vessels, which were now enlarged, particularly the inferior thyroid artery.

"The redness of the skin, however, continued to increase, and that of the scapular portion of the tumor to become more and more of a purple colour, till at last ulceration commenced on the most prominent part. Several considerable-sized portions of coagulated blood were discharged, along with some healthy pus, through the ulcerated opening; and, on the twentieth day after the operation, the ulceration of the integuments had closed, and nothing of the tumor remained but some wrinkling of the skin, and a considerable degree of thickening of those parts on which the base of the tumor had rested. These continued to diminish, and, at the end of the fifth week from the time of the operation, the neck had nearly resumed its natural form, a slight degree of inequality only remaining; the ligature had come away; and the patient's general health (to the management of which the greatest care had been bestowed,) appeared now to be completely re-established." (P. 220 —224.)

The author thinks it possible that this operation may, under particular circumstances, be preferable to tying the ligature between the aneurism and the heart, even when that operation is practicable. He regards it, however, as indispensable to success that there be no vessel, arising either from the sac itself or from the artery between the sac and ligature, sufficiently large to keep up the circulation of the blood through these parts. To ensure this, the ligature ought to be applied as close to the tumor as possible: he conceives that there may be cases wherein it might be practicable to tie separately any branch which intervened.

### 16. *On the Medical Properties of the Subcarbonate of Iron.* By Dr. Elliotson.

Dr. Elliotson has devoted considerable pains to ascertain the operation of certain remedies, having shown the efficacy of prussic acid in affections of the stomach, and the inefficacy of antimonial powder, as usually administered. On the present occasion he directs our attention to the subcarbonate of iron, the preparation which has been so successfully employed by Mr. HUTCHINSON, of Southwell, in diseased conditions of the sentient system: and here we must observe, that the author has evidently mistaken the extent to which this gentleman has exhibited this remedy. He says, "Dr. Hutchinson, who has written upon the virtues of this medicine in neuralgia, and probably employed it more than most others, tells us that the greatest quantity he has ever found necessary is ninety grains in the twenty-four hours. This mode of expression leads to the supposition that this is the largest quantity he has ever given." Now, in the very first case given in Mr. Hutchinson's Essay, the dose is stated to have been a drachm three times a-day, which makes 180 grains in twenty-four hours; and we know, from correspondence with Mr. Hutchinson on the subject of his treatment of neuralgic affections, that in many instances he has given to the extent of two drachms, and two drachms and a half, three times, and occasionally even four, times a-day. We make these remarks, not as detracting from the merit of Dr. Elliotson, who states that it had for many years been his custom to prescribe the medicine in drachm doses,—but as advocating the claims of Mr. Hutchinson to the gratitude of the profession, for having been the first to lead them to the full and free exhibition of the subcarbonate of iron in neuralgic diseases. This practice, we believe, has been very extensively adopted : we have ourselves repeatedly pushed the medicine to the extent of four scruples three times a-day, with success; and, in a case of recent occurrence, which we hope to lay before our readers in a future Number, we know that it was carried to the extent of half an ounce three times a-day.

Dr. Elliotson, however, goes further than this; he remarks—

"But I have ascertained, by very numerous trials, that, when the use of the medicine is proper, it may be given in far larger quantities; that two, three, and four drachms may be given every six, nay every four hours; that this dose of half an ounce may be at once commenced with, and does not require, in order that it may be borne, to be reached by slow degrees, and that it may be continued many weeks without the slightest inconvenience.

"I have become acquainted with these facts during the last twelve months. It had for many years been my custom to prescribe the medicine in drachm doses; but having, about a year ago, several cases in which its exhibition appeared proper, and never having observed any sensible effect from it, I began to think that a drachm was probably not the utmost quantity which the stomach would bear, and resolved to ascertain, by cautious augmentations of the doses, how much might be taken. It soon became evident that there was no occasion for timidity, and my uniform experience in nearly a hundred cases enables me to present the results just stated. When the medicine is proper, I believe there is generally no limit to the quantity which may be taken, except the unwillingness of the patient to swallow it, and the inability of the stomach to manage so heavy a mass. Neither headache, thirst, heat, foulness of tongue, griping, nor constipation, has occurred;* nor been increased, if already present. Nay, pain and heat of the head and giddiness existed already in some cases, and ceased during its employment.

"I am enabled to say that these quantities are ordinarily borne, exactly as is the case with the pulvis antimonialis, not because the operation of the medicine is resisted by an unnatural condition of the system, not because the quantity is gradually reached, and not because the article is bad. The article was procured from a variety of druggists, from many of the most respectable houses in London, and was in fact, in several examples, known to be a genuine article, prepared according to the letter of the Pharmacopœia.†" (P. 235—237.)

This augmentation of the usual doses of iron is not confined to the subcarbonate, as the author informs us that a scruple of the sulphate made into pills with the extract of gentian, is borne just as well as half an ounce of the former; and that he himself took a drachm of green vitriol daily for several weeks, without any sensible effects.

One case of *paralysis agitans*, and nine of *chorea*, are related, in all of which the iron proved successful, although in several instances symptoms were present, which we have been taught to regard as contra-indicating this medicine,—viz. headache, vertigo, and plethoric habit. Three of these we subjoin in illustration.

*Paralysis Agitans.*—"April 17, 1823. Joseph Deacon, aged twenty-eight years, ill one year. Constant shaking of the legs and arms. The intensity of the tremor varies. Till within the last week, the agitation would sometimes cease for a few hours, or even a whole day, but for the last week has been constant. At first, sometimes only one leg was affected, sometimes both. He has pain in the head, loins and legs, and vertigo, and cannot fix his attention.

"I at first brought out and maintained a copious crop of pustules on his legs, by the ointment of tartarised antimony.

* "I wish not to assert that such inconveniences will never occur; but the absence of them in all my trials justifies me in believing that they will not prove common occurrences, with even half-ounce doses. Many practitioners are no longer deterred from giving iron by headache, foulness of tongue, &c. if other circumstances indicate its employment. I believe that, when it excites and disturbs, this arises from a peculiar susceptibility to its influence, (as I shall endeavour to prove with respect to the oil of turpentine;) unless, of course, the system is already in a highly excitable state.

† "If the articles used by me were all proved to be bad, the importance of my facts would remain little diminished, (and the same would hold in regard to the pulvis antimonialis,) because the variety and respectability of the houses at which the medicine was purchased shew that the facts, if not true of genuine subcarbonate of iron, are true of the subcarbonate in common use."

"He next took one ounce of oil of turpentine, and then two ounces every other day for above a week.

"He then went through a course of sulphate of zinc, taking at first one grain, and at length seven grains and a half, every six hours; but the dose was reduced to six grains, on account of nausea. During this course, his head was kept constantly wet with a cold spirituous lotion; he was cupped on the occiput; had a blister on the forehead, and another on the occiput, kept open nearly a month with sabine ointment; and eight leeches applied daily to the temples, from the 20th to the 31st of May; and took frequent purgatives: yet he was no better.

"June 3d.—I prescribed half a drachm of the subcarbonate of iron every eight hours, with eight leeches to the temples every other day; and a perpetual blister to the forehead, on account of the violent pain of the head, the vertigo, and the heat and smarting of the eyes.

"June 7.—He felt already better.

"June 11.—The dose was increased to one drachm, and the leeches continued.

"June 14.—One drachm and a half for a dose.

"June 17.—Still better in regard to the shaking; but the headache, vertigo, &c. as before.

"June 21.—Two drachms for a dose. Another perpetual blister to the forehead, the last having healed.

"July 1.—He felt so satisfied that neither the blisters nor leeches had procured the least relief to his headache, vertigo, or the pain and smarting of the eyes; and these symptoms had so clearly not been aggravated by the iron, that the former were discontinued, and the dose of the latter augmented to two and a half drachms three times a-day.

"July 8.—Three drachms for a dose. The shaking was lessening rapidly, as well as the vertigo, headache, &c.

"July 12.—Still better. Three drachms four times a-day.

"July 15.—Scarcely any shaking observable. No headache, vertigo, or any other unpleasant feeling about the head.

"Finding himself so well, he left the hospital, by his own desire, July 17th; taking with him a sufficient quantity of his medicine to last a month. I saw him several months afterwards, perfectly well." (P. 240—244.)

"Case IV.—April 8, 1824. Elizabeth Powell, aged twenty-two years, ill with chorea six months. Had the disease six years ago. The right side most affected. Pain of the forehead, especially on stooping. Has been bled and purged without benefit.

"Two drachms of the subcarbonate of iron every eight hours, were ordered.

"On the 13th she was rather better, and on the 17th much better. Her speech was greatly improved, and she could hold any thing in her right hand. The pain of the head was quite gone. She afterwards complained of pain in her left side, which was removed by two bleedings. She was dismissed on the 18th, perfectly well.

"CASE V.—Aug. 12, 1824. Elizabeth Howel, aged eleven years, affected with chorea five weeks. Her sister, thirteen years old, had laboured under it two months. She has a florid complexion, and is in high condition. The right side is much more affected than the left. Occasional headache; the bowels usually costive.

"She was ordered one drachm of the subcarbonate of iron every six hours.

"On the 31st, the dose was increased to three drachms; and, on the 18th of September, to half an ounce.

"Sept. 21.—She was no better, (the weather had been for some time intensely hot, and had rendered her irritable;) and for some reason, I forget what, the sulphate was substituted for the subcarbonate, in doses of five grains three times a-day.

"Oct. 2.—The dose was advanced to seven and a half grains; and, on the 5th, to ten grains.

"Oct. 9.—She was no better; and, being satisfied that the subcarbonate would cure her, I resumed it. She began with two drachms every six hours; advanced, on the 13th, to half an ounce; and, on the 23d, the same dose was taken every four hours.

"She had mended rapidly for some time, and was dismissed perfectly well on the 4th of November. The disease returned in a very slight degree, but yielded permanently to the medicine." (P. 247—249.)

An interesting case of *Hydrophobia*, by Dr. GREGORY, comes next in succession; but our limits compel us to decline giving it. The author is inclined to regard hydrophobia as belonging properly to the genus Cynanche; an idea which many of the phenomena render extremely plausible. He states that there is a premonitory period of two or three days, and occasionally longer, during which remedies might be applied with more prospect of success than when the disease has become developed.

Our readers will find, in the Foreign Department of the present Number, a curious document by Dr. MAROCHETTI, which contains all that is at present known or conjectured about the sublingual pustules, said to occur as an intermediate link in the chain of events between the reception of the canine virus and its hydrophobic influence upon the constitution.

### DIVISION II.

### FOREIGN.

ART. III.—*Observations sur l'Hydrophobie, indices certains pour reconnaître l'existence du Virus Hydrophobique dans un individu, et moyens de prevenir la developpement de la maladie en detruisant le germe, suivés des principaux ces pratiques, depuis l'année* 1813 *jusqu'en* 1823. Par le Dr. M. MAROCHETTI, Medicin et Chirurgien à la grande Amirauté de St. Petersburg, Membre Correspondant de l'Academie Royale des Sciences de Turin, et Membre de la Societé Physico Medicale de Moscoue, Chevalier de l'ordre de St. Anne de Russie.

*Observations on Hydrophobia; with certain signs by which the existence of the Hydrophobic Virus may be known in any individual, and means to prevent the development of the Malady by destroying its germ, followed by the principal Cases from the year* 1813 *to* 1823. By D. M. MAROCHETTI, physician and surgeon to the Admiralty at St. Petersburg, &c. &c.

So much has lately been said respecting hydrophobia, and instances of that disease have appeared in some countries, at least, to be so much upon the increase, that we conceive we shall be rendering an acceptable service to our readers by laying before them the researches of the author, the title of whose work appears at the head of this article, and whose peculiar opinions have been frequently referred to in detached articles of intelligence from time to time.

In presenting the contents of this work to the consideration of our readers, we by no means wish it to be understood that we participate in all the sentiments of the author, nor are we prepared to assent to all his doctrines; but, at a time when the very existence of the disease treated of is attempted to be denied, we think, that a detail of what Dr. Marochetti has to urge in support of the opposite doctrine, cannot fail to be received with attention. Passing by a page or two of introductory matter, we commence with the Theoretical Observations on the Hydrophobic Virus.

"Hydrophobia, (says our author,) the terrible consequence of the bite of a rabid animal, is amongst the accidents to which humanity is exposed,—a disease, so much the more cruel, because all the means hitherto employed to save the victims appear to be inefficient; so that, notwithstanding a few exceptions, all unprejudiced practitioners agree in declaring, that no specific against this poison exists, when absorption has taken place and the symptoms are declared. I am (continues Dr. M.) of the same opinion, but I will take upon me to say, that, with a knowledge of the cause, it is possible to prevent the evil; and that, consequently, it is useless to search for a specific; since, according to the old adage, 'ablata causa, tolitur effectus.' In spite of the researches made by medical men in all climates, the real seat of the disease was unknown until the year 1820. A crowd of medicines has been employed, some no doubt because they have succeeded, or appeared to succeed, in some one particular case, in which, in truth, the disease did not exist; others have been administered simply as preventives. But the point most essential to be known, is the cause of the disease; its effects are, unhappily, too evident. In this memoir, the author proposes to point out, 1st. What he has himself observed. 2nd. The origin of the discovery. 3d. The preservative cure; and, lastly, the different pathological conditions which he has had to treat, as well as the knowledge he has acquired by practice.

"Having passed nearly eight years in the southern governments of Russia, where there is a great number of dogs, and where hydrophobia is very frequent, I have (says our author,) had the grief, upon a great many occasions, to see many of these victims perish under my eyes; and, of necessity, was induced to make many researches relative to this disease, and to try all the known means of cure. Situated in a village in the midst of these unfortunate people, some bitten by mad dogs, others by rabid wolves, the doctor was peculiarly enabled to observe and to follow the course, and trace the progress of this horrible malady. The following is the result of his observations:—1st. He was convinced by experience, that when several persons are bitten one after another by a rabid animal, that the first exhibits symptoms more alarming and severe than the second, the second more than the third, and so of the rest, the virus always acting in an inverse ratio with respect to the number, so that the tenth or eleventh person may be considered as free from danger; and this is found to be sometimes the case. 2nd. The virus does not reside always in the throat of the animal, it is only accumulated there at the expiration of a certain time; the bite cannot be considered poisonous in this interval: here, then, are two circumstances under which the animal cannot communicate the disease. 3. A profuse suppuration ensuing in consequence of an abscess, or from a large wound, commonly extinguishes the virus; and the person, who is in this condition, has not much reason to fear the attack of the disease; though this may not always be the case. 4. The hydrophobic virus does not, like the pestilential miasma, lose its intensity by being communicated from one person to another; but, in proportion to its quantity, it acts with a greater or less degree of violence, and it is precisely owing to the different dose of the virus, introduced into the wounds made by the bite, that the symptoms are developed at different epochs: unhappily, the effect is not less fatal, although sometimes more slow than at others.* 5. It is certain, that the poison does not long remain in the parts bitten, but that it is carried entirely (*dans toute son integrité*) to a part of the body which will be designated by-and-bye; and that, in that part, it acts as a most powerful astringent. The poison, in thus accumulating, inflames or irritates, and shuts up those channels by which nature attempts its expulsion from the animal economy. 6. There exists only one means of preventing the development of hydrophobia in the individual who is about to be the subject of it; that is, to evacuate the hydrophobic poison as soon as it presents itself. It becomes, therefore, incumbent upon us next to point out where it resides, and how to evacuate it. The

* We do not pretend to reconcile this passage with a preceding one that appears rather to contradict it, but we give it as we find it in the original.—Edit.

sublingual glands are two in number, one on each side under the tongue, between the genio-glossi muscles. These glands give rise to two or three secretory canals, which open into those of the sub-maxillary glands, and these open one on each side of the frœnum of the tongue. It is precisely to this point, at the extremity of these canals, that, after the bite of a rabid animal, and at the expiration of a certain number of days, the hydrophobic poison is carried, and that it is there retained temporarily, forming towards the two points which have just been indicated one or two little tumours, or vesicles of an unequal size; sometimes they present the appearance of a fleshy purse, at others they form a red equal tumour, and sometimes they are met with in the form of little unequal excrescences, of a brownish red colour. By examining them with a probe, a fluctuation is occasionally perceived in them, which is, as is proved by experience, the hydrophobic poison; it is there that nature delivers the enemy to us, and it is from thence that we must expel it. It is not possible to declare exactly the epoch in which these little tumours become evident; commonly the period when they may be expected to form, is from the third to the ninth day after the bite: if the virus is not evacuated within twenty-four hours, it generally disappears by re-absorption, (although two exceptions to this rule are mentioned,) and then no traces of its previous existence are left. A double metastasis appears to take place towards the brain, after the disappearance of these tumours, the symptoms of hydrophobia begin to manifest themselves by degrees, till it reaches its acmé, and the patient dies in a paroxysm. On opening the body, no traces of the ravages of this cruel poison can be found, which proves incontestably the *necrotic* action of this poison operating directly on the brain, since every other cause of death, which exercises its action upon any other part or organ, leaves traces of its operation. Pathological anatomy, in spite of every research, has discovered no sign capable of fixing the attention of physicians, or explaining the effects, because the cause was unknown; that is to say, the re-absorption of the poison.

" *On the Means of Cure.*—The first thing to be done when a person believes he has been bitten by a rabid animal, is to immediately apply a blistering plaster to the bite, if it has not been possible to employ a caustic at the very moment; in either case, a blister ought always to be employed. The part where the tumours are formed are to be examined as soon as possible; this examination ought to be continued for forty-two days once, or even twice in the day, for greater security: if at the end of this time these tumours are not perceived, one may be persuaded that the person was not infected with the hydrophobic poison, especially if the patient has not made use of the decoction, as will be afterwards mentioned; but, if it has been employed in the beginning, and the tumours do not make their appearance, then, if we know that the animal was really mad, we may be persuaded that the virus has been destroyed by the remedy. Whatever may be said as to the virtues of the plant, Dr. Marochetti is convinced, that it is a real preservative from this malady, since it has been found alone capable of neutralising the poison in many cases. The fact is, that where it has been possible to exhibit the decoction from the period of the bite, the tumours have seldom been found. During the examination for the discovery of these tumours, they must, as soon as they are perceived, be opened by a very sharp lancet, or with curved scissors; and they must afterwards be cauterized, by applying immediately upon the spot a small button cautery, similar to those used by dentists to destroy caries of the teeth. But as it is sometimes difficult to make the incisions into these tumours, either from the want of proper instruments, or when the patient has an invincible repugnance to a cutting instrument, or when the swellings are of a very small size, then they may be cauterized, taking care, however, to destroy them entirely. This operation is performed, by raising up the patient's tongue with one hand, when he either cannot, or does not know how to elevate it towards the palate; it ought to be covered with linen, and raised up, and a little on one side, in order that the tumour may be perfectly in view, and exposed to the instrument; to facilitate cauterization, it may be held by an assistant: as soon as the patient is placed in a proper posture, as many longitudinal incisions may be made as there are tumours, making the patient afterwards wash out his mouth, and then the cautery is to be applied. If these tumours or pustules are very apparent, sometimes a drop of sanious lymph escapes, (which the author once saw.) In every case after the application of the caustic, the patient must be made to drink a decoction of the whole plant of the genista lutea tinctoria, prepared in the proportion of one ounce to two pounds of water boiled to half the quantity. It is necessary to explain, that the decoction ought to be prepared and administered before the operation, and its use continued during the six weeks that the patient ought to be subjected to examination and treatment. The dose of the decoction is two pints in the day, and even more if the patient can employ it as a common drink; it ought to be taken always warm. The powder of the leaves and flowers of the same plant ought always to be employed internally at the same time, in doses of from two to three drachms in the day, at three or four times, regard being had to the age and constitution of the patient. It produces no apparent effect on the economy; and, consequently, it may be given even in larger doses, when it does not excite vomiting; the powder may be given either in the decoction or in water, but not mixing easily with liquids, patients often find some difficulty in swallowing it: in that case, the best plan is to exhibit it dry, sprinkled over bread. It appears even to have more virtue when administered in this manner.

The operation of opening and destroying the pustules is so easy, that whoever has once seen it done, will be able to perform it; not only professional men, but others may, in case of necessity, undertake it without fear. With regard to the wounds caused by the bite, a blister is always to be applied to them, and suffered to remain for twenty-four hours; then the blister, that has been formed, is to be removed, and if the surface is not very much irritated, a second blister is to be applied, and suppuration is to be kept up by some digestive, or by the blister plaster itself; milder means are not used, unless the inflammation should be very great, although some degree of irritation in the wound is desirable; this suppuration ought to be maintained for six weeks. When the wounds are of a severe nature, and assume a bad aspect, or the suppuration is of a bad kind, a cataplasm may be applied, composed of the residuum of the decoction. It is often necessary to administer a purgative to the patient once in a week; and sometimes, also, an emollient clyster: by these means, the constipation caused by the *tannin* of the plant may be obviated. It is highly essential to pay attention to the appearance of the precursory symptoms of the formation of the sublingual tumours or pustules. The following are the chief of these: towards this period the pupil is dilated, and becomes fixed; the look is melancholy; the patient is restless, and experiences pains in the head, of a greater or less degree of severity. These are the only premonitory symptoms observed, and they almost always precede the development of the disease.

### *Pathological Observations.*

"*The Discovery of the new Method of Treatment.*—When Dr. Marochetti was residing in the Ukraine in the year 1813, in a small village called Rijarka, one evening in autumn, at the hour when the peasants returned from their labours, a large dog affected with hydrophobia, belonging to a neighbouring village, bit fifteen persons of all ages and both sexes; the doctor, being at some distance, was only informed of this the next morning; on visiting the village, he first fixed upon a large house wherein all these unfortunate people were assembled, and people were appointed to take care of and wait upon them; in the mean time, a deputation of old men waited upon the doctor, begging him to suffer these poor people to be treated by a peasant in the vicinity, who had, for a long time, devoted himself to this occupation with constant success; this man was a Cossack, whose family had made their living by treating persons bitten by rapid animals for a number of years, from father to son, without communicating their secret to any one: these old men assured our author, that they could testify that the plan pursued by this peasant had saved the lives of some hundred of persons. Dr. Marochetti had before heard of this man, and was anxious to be convinced of the efficacy of a plan, of which he could form no idea; for this purpose, after having obtained permission from the lord of the village, he permitted the countryman to treat the patients but on two conditions: first, that he should be present during the whole process; and, secondly, in order to be assured that the dog was really rabid, that one of the bitten persons should be treated by the methods usually employed professionally upon these occasions. A little girl, six years old, was therefore selected from among the number for the purpose of undergoing medical treatment, the cauterization of the wounds not being neglected; calomel, camphor, the alisma, (plantago,) and opium, were prescribed for her; the others began to take the decoction of genista tinctoria, which the peasant made in the presence of the doctor, who did not recognize the plant at the moment; but, upon comparing some portions which he carried away with him, he found its description in Linnæus. In the Ukraine, it goes by the name of *drokdrok:* it is used in that country as a yellow dye. Passing the greater part of his time with these patients, he committed his charge to a surgeon during his absence, who was ordered to prevent the countryman from acting when the Doctor was not present. This man began by looking under the tongue of each individual, night and morning. As the pustules made their appearance, he pointed them out to the author, opened and cauterised them; afterwards, he caused the mouth to be washed out with the decoction above mentioned.

The little girl, in whom the method of treatment had been followed with the greatest exactness, and conformably to the plan usually recommended in these cases, fell a victim to this experiment. On the morning of the seventh day after the accident, she was all at once seized with the symptoms of hydrophobia. Eight hours afterwards, she died in presence of the Doctor, in a paroxysm of convulsions. Our author could not say whether this girl had the pustules or not, because he paid no attention to this circumstance, which he now regrets very much.

Of the fourteen persons who remained, twelve underwent the operation of having the tumors opened, and were saved; two others, who perhaps had been last bitten, had no appearance of pustules, and were also saved. All the above individuals, after having made use of the decoction of genista for six weeks, their wounds (which had been kept in a state of suppuration the whole of that time) being cicatrised, were discharged, quite well. Having resided for three years after in this village, the author saw these same persons many times subsequently, and therefore was convinced that the cure was perfect.

The above cases were so decisive as to the efficacy of this method, that there was scarcely a necessity of proving it further; but, as the design was to convince others, the Doctor was determined not to give publicity to them until confirmed by subsequent observation: and, unhappily, opportunities occurred but too often.

Being at Podolia in 1818, and inhabiting a small town called Meskovka, in the month of February, twenty-six persons, Christians and Jews, were bitten by a rabid dog. This animal made incursions into the village, and bit all the persons he met with; he then concealed himself, and again repeated his attacks. At length the inhabitants assembled, and searched for him in his usual place of retreat, and they found him lying dead near a heap of corn. In spite of all the endeavours of our author, he was unable to discover the exact order in which the people had been bitten. So considerable a number could not be shut up in the same house, and they were therefore obliged to be separated into three divisions, in as many different houses: in the first, nine men were placed; in the second, eleven women; in the third, six children; and in each of these houses a Jewish surgeon was placed, to prepare the decoction, to see it properly taken, and to make an exact report of every thing that had taken place during the night.

The result is thus related:—In the first division, five persons had the tumors under the tongue; in the second, the whole number; and, in the third, only three of the children. Of all these individuals, those who had severe wounds, and the greatest number of them, had the tumors on the third day; others on the fifth, seventh, and ninth days; and one woman as late as the twelfth day after the accident. This woman had been bitten very slightly in the right leg. The seven persons who had no appearance of pustules drank nevertheless the decoction of genista during six weeks, and were liberated from their confinement along with the others, excepting those whose wounds were not healed at that period.

At different periods, and in different places, during our author's residence in the Ukraine, he had occasion to treat, besides the above-named individuals, six persons in the same manner, and with equal success; among the rest a peasant, who had been bitten by a mad wolf in passing from one village to another. His cries brought other men to his assistance, who helped him to kill this ferocious animal, which was known to be hydrophobic. The only difference in the cure of this man was that, among other wounds, he had been bitten at the fore part of the articulation of the tarsus, and the tendons and articular ligaments had been so torn, that all the means employed could not prevent a great deformity of the foot. This man was confined to bed for two months.

Dr. Marochetti next proceeds to the relation of the most interesting cases of this kind which came under his observation at Moscow, when he was attached to the Galatzin hospital, which he quitted in February 1824, for his present situation.

In 1820, on the 13th January, two house-dogs, one of which was chained up, belonging to a citizen named Peteroff, were bitten by a strange dog labouring under hydrophobia, and which escaped immediately. Their master, who had heard the noise in his room, took no notice of the event. On the 24th of March following, this man's daughter, ten years of age, going into the court-yard early in the morning, before it was quite light, was suddenly bitten in the posterior part of the thigh and in the right knee, by the dog which was at liberty, and which had not returned to the house since the preceding day: neither had he nor the other dog eaten or drank any of the provisions which were daily given to them. The father, struck by the cries of the child, arose, and, running to the spot, found her in a state of great alarm, and complaining of severe pain in the wounded part. The dog which had bitten her was standing beside her in a state of stupidity, his eyes glistening, the tail depressed, and foaming at the mouth. Peteroff, recollecting what had happened on the night of the 13th Jan. and remembering that for some days past the dog had not approached him as usual, he did not doubt that the animal was rabid, and therefore shot him. His residence not being far from the hospital, he went to consult our author on the same day. After having examined the wounds, which were not very deep, though lacerated, and producing much pain, in consequence of some of the fibres of the aponeurosis of the fascia lata having been torn, as well as the integuments of the knee, notwithstanding the pain, a blister was immediately applied to the wounds; and, as the patient did not then remain in the hospital, she was brought there again on the following day. Two ounces of the genista were given to the father, explaining to him the manner of making the decoction, and he was ordered to bring his daughter to the hospital every day at the same hour. No intelligence, however, was obtained of her for three days; but, on the morning of the fourth day (the 27th March), Peteroff came to the hospital with his daughter, and informed the Doctor that he had been obliged to kill the other dog, who showed a disposition to bite him as he went to him with his food: observing that his chain had not touched the water, which was close to him, that he foamed at the mouth, and his eyes were fiery, he did not doubt that he also was affected with the hydrophobia. On examining the mouth of the girl, it was found that the pustules were formed, and even of a duller colour than ordinary; a proof that they had existed from the previous evening. The patient was admitted into the hospital, and Dr. Damon, the chief physician of the hospital of Peter and Paul, was invited to see her, in consequence of a request which he had made; the pustules were opened in his presence. The unusual colour of these pustules gave our author some uneasiness,—thinking that their destruction might be too late, and that a portion of the poison might have been re-absorbed; for, on examining the parts beneath the tongue the evening of the same day, the sublingual glands were covered with a kind of miliary eruption, of a yellow colour, in the form of almost imperceptible vesicles, containing a matter similar to a very thin pus. Without being able positively to say that this was the hydrophobic virus, Dr. Marochetti took the precaution to cauterise these pustules lightly with the lapis infernalis, in order to prevent the fatal result that might have ensued. A piece of rag soaked in oil was placed upon the part, in order that the action of the caustic might be arrested at the surface. A little departure was made from the common mode of treatment, by cauterising the bites, in consequence of their appearing of a deep yellow colour.

As this case presented phenomena which were not met with in any other, it is essential to mention every circumstance that can tend to clear up the obscurity existing with respect to the disease; the detail is continued in the form of a journal.

On the 28th, nothing particular occurred; the patient was tranquil, ate, drank, and slept well.

The 29th, that kind of eruption which had appeared under the tongue on the 27th, was again seen in some degree, and treated in the same manner. The patient was weak, felt violent pains in the head; all the external glands, as the parotids, submaxillary, axillary, and inguinal, were considerably swollen; there was besides an accession of fever towards the evening, which lasted nearly three hours. After this she became calm, and slept well.

On the 30th, in the morning, she was tolerably well; but in the afternoon she felt hot and restless, with an unequal and frequent pulse. In bringing the glass to her mouth for the purpose of drinking, she was seized with an universal trembling; but this was only momentary, she afterwards drank without repugnance.

The 31st.—Although the glands were in the same state, she made no complaints. Some small yellow spots had appeared under the tongue: they were cauterised, which neither prevented her from taking her nourishment nor medicines.

April 1st, the ninth day after the bite, she was well during the whole day; but, towards eleven o'clock at night, awoke in a start, cried out several times, and on a sudden jumped out at the bottom of the bed, grasping firmly with her hands the board containing the name and disease of the patient; afterwards she fell upon the floor, being awoke by the pupil who watched her. At the commencement of this attack, our author was close to her, and found her in a state of insensibility, cold, the pulse changed and hard. This condition continued until the morning.

2d.—She continued tolerably well till two o'clock in the afternoon; she was then seized with a trembling of her hands, but took the decoction without repugnance; sat upon her bed without speaking, and looked at the surrounding spectators with haggard eyes, but without making any reply to the questions proposed to her. Her countenance had a yellow tint, changing colour every moment, and she was in a state of stupor; the pulse was full and frequent. This condition only lasted twenty minutes; then she returned to her usual state, asked for some drink, which she swallowed with avidity, and slept until the morning.

These frequent changes and different symptoms caused our author to reflect that the ordinary dose of the genista was, perhaps, too small to destroy that portion of the virus which might have been re-absorbed in the brain or circulating in the blood; and therefore, wishing to produce a more prompt, as well as a more efficacious, action of the remedy, he determined to give it also in powder, in the dose of three drachms in the day, divided into three portions, sprinkled upon slices of bread; and this was continued half through the cure. It has often been remarked, that nature points out to the sick many substances or aliments which medical theory would forbid the use of: the same may be said of the genista; for it was observed that all the bitten persons not only became accustomed to it, but actually took it greedily, although the taste is far from agreeable.

On the 3d, the patient was well the whole day, ate with appetite, and slept soundly.

The 4th.—Up to eleven in the forenoon, nothing particular occurred; then she became silent and abstracted; but, after five, she was again tranquil, though she complained of a dull pain, and a kind of uneasiness in the swollen glands.

Up to the 9th, no remarkable alteration is noted.

On the 10th, she was well until noon. After dinner she appeared dull, though complaining of no pain. The inguinal glands of the right side enlarged suddenly; a tumor, the size of a large bubo, appeared without any preceding inflammation. This impeded her walking. The enlargement of the other glands began to diminish, and in the space of twenty-four hours disappeared. At seven o'clock, the pulse was greatly agitated; she was hot and restless: her senses were, however, perfect, although her look was ferocious, and she did not sleep in the night.

The 11th, at eight o'clock in the morning, she was in the same state. She was very much constipated, and the belly swollen. Four ounces of a laxative infusion was given: she was purged but little, but was much better afterwards.

12th.—She went on favourably; the enlargement of the glands was diminished.

13th.—She continued to improve. The same on the 14th; but again the swelling of the inguinal glands increased; and then, in order to assist nature in expelling some morbific matter, an ammoniacum plaster was placed upon them. There was, however, no fluctuation in the tumor.

17th.—She was hot; the swelling became very painful, and there was fever in the night. She ate but little during this day; but she drank her decoction, and took her powder as usual.

18th.—In the morning, the patient had a hard and frequent pulse; the eyes wandering, the pupil very much dilated; with great palpitation at the heart. Whilst drinking the decoction, she had an universal trembling; was very pale all the day; but got better towards evening, and slept all night.

From this time, the tumor, to which the plaster was continued to be applied, diminished little by little, and at the termination of the cure was quite resolved. She was purged three times, and went on with little alteration; till, at length, on the 2d May, she quitted the hospital perfectly well.

This patient was seen at the end of a twelvemonth, in the enjoyment of good health. The alarming symptoms (says M. Marochetti) which appeared so many times during the cure, and which were conquered by the virtues of the remedy, claim the attention of practitioners, and demand an exact use of this precious means of cure from all the friends of humanity.

In a village in the neighbourhood of Moscow, a countryman, named Mironow, was bitten, on the 19th May, 1821, by his own horse, attacked with hydrophobia at the moment when he was harnessing him to his cart. This horse, after biting its companion, bit the above-named person on the hand, producing a considerable contusion and laceration; and, after running from place to place, without however going to any distance, biting the earth at every step, he suddenly fell down dead. The other horse was killed before any signs of hydrophobia appeared. Mironow was taken to the hospital on the 23d, five days after the accident, having applied a blister to the wounded part: the under part of the tongue was examined, but nothing was perceived. Three drachms of the powder of genista were given every day in three doses, as well as two pints of the decoction. Having been admitted early in the morning, he suffered nothing until about four o'clock in the afternoon; but he then complained of palpitation of the heart, and an inclination to vomit. This lasted about an hour, and it was supposed to have been produced by the powder and the decoction. He afterwards supped, and slept well. The sixth day he went on favourably, until two o'clock P.M.; but soon after he became hot, complained of headache, of nausea, and was very feeble; the pulse was full, and irregular. In about an hour he felt better, and passed the night well. The seventh day, the pustules were discovered under the tongue; but, as they were not very apparent, they were not incised and cauterised until three o'clock. After this he went on well till the ninth day, at five in the evening, when he experienced violent pains in the head. On examining under the tongue, some portions of the tumor were thought to remain, and therefore they were cauterised again, limiting the action of the caustic by means of the oil. From eight o'clock of this day until the 12th, nothing remarkable appeared: on that day, being constipated, he had much pain in the head; but, being well purged, and continuing to take the decoction, he felt no other unpleasant symptom, and quitted the hospital on the forty-third day, quite cured.

At this period Dr. Marochetti became convinced that the cauterisation of the tumors was more essential than their incision; and, consequently, he urges those who employ this treatment not to omit the use of the cautery in every case, employing a large needle heated to redness, in case they are not provided with the button cautery, which he now employs generally.

On the 8th September of the same year, three domestics of Prince Galitzen were received into the hospital, having been bitten in the same day by a rabid dog. Two of them were bitten in the hands, and one on the nose. They took the genista, both in powder and decoction, for six weeks; suppuration of the wounds being kept up. No symptom of the disease appearing, they quitted the hospital quite well. In these cases there were no pustules, and therefore it is to be presumed that the remedy was alone sufficient to destroy the virus, since the dog was certainly rabid.

On the 3d of November, 1822, a girl, fifteen years of age, covered with a pelisse formed of a sheep's skin, whilst passing through the street from her own house to a neighbour's, was attacked by a dog, who, not being able to find any part exposed to his teeth, threw the girl down, and jumped upon her face. Having her mouth open, crying for help, he bit her in such a manner as to tear away three of her teeth, broke the alveolar processes, tore the upper part of the cheek so as to expose the maxillary bone, and at the same time divided the ala of the nose on the left side, burying his teeth into the substance of the jaw-bone. Her brother, eighteen years of age, attracted by her cries, and endeavouring to force the dog from his sister, was bitten on several parts of the hands. The surgeon of the place, not knowing what to do on this occasion, sent these unfortunate people into town, in order to await the orders of M. Clement, the director belonging to the palace of the Prince, who sent our author to the hospital, in order to examine their condition. A wound so extraordinary as that of this young girl left but little hope of being able to save her; for a wound in the mouth gives rise to an immediate absorption of the virus; but, not being willing to omit any means, however uncertain, and having made such applications as were possible under the circumstances, the concentrated decoction was prescribed,—the powder could not be given. To the brother the blistering plaster was applied. They were then sent back to the house of the master, the surgeon being instructed in what manner they were to be treated; the Doctor proposing to visit them every day. In spite of every attention, however, the bites of the girl assumed a gangrenous character. By employing proper means, suppuration became established. The sublingual pustules, if they took place, could not be seen on account of the enormous swelling of the interior of the mouth, as well as of the whole face, leaving only, as it were, a narrow passage, through which the patient breathed, and could drink, though with some difficulty. A slow fever continually tormented her; she had frequent paroxysms, with vomiting, pains in the head, tremblings, and universal weakness. In spite, however, of the deplorable condition in which she was,—not being able to swallow any solid nourishment, and being in a state of continual insensibility,—she lived till the thirty-eighth day. Until then she had no hydrophobic symptom, but at this time they became developed with an extraordinary degree of malignity: she died at the end of fifteen hours, in terrible agony.

The brother, who was placed in the same room, and who regularly took both the powder and decoction, and whose wounds were treated in the usual manner, had the tumors under his tongue the seventh day after the bites. Having incised and cauterised them, there was from time to time changes in the condition of the patient, but no alarming symptom; so that, after the forty-third day, he was liberated in good health, which he still enjoys.

On the 25th April, 1823, a citizen of the town of Nejia, was admitted into the Galatzin Hospital, fifty-five years of age. This man, going from his house to the cellar, was bitten, about nine o'clock in the evening of the 24th, on the side of the left thigh, by a strange dog in a state of hydrophobia, who had bitten three other dogs belonging to the house the day before, and had afterwards concealed himself. This man was scarcely admitted into the hospital, before he complained of nausea and pain in the head; the pulse was frequent and agitated. He was made to drink of the decoction immediately, but he vomited it; and this happened repeatedly. On the third day, the pains in the head were more violent, and there was some fever. This day the three dogs became affected with hydrophobia, and died in the night. On the fourth day, the vomitings were less frequent, and the headache diminished; and, as the decoction of the genista was not retained on the stomach, the powder was administered upon slices of bread, in three-drachm doses in the day. This was not rejected, and was continued. The following day, the fifth from the accident, he was restless; he vomited twice, and the pulse was agitated. In the evening, the man had much fever, with heat and vomiting; the eyes were haggard, the pupils greatly dilated; the wounds caused by the bite were of a deep yellow colour. Two pustules, larger than ordinary, were formed under the tongue; they were taken away with the scissars, and cauterised, as were the bitten parts also. As soon as the pustules were operated upon, all the symptoms disappeared with an extraordinary degree of rapidity, so that, in less than a quarter of an hour, the patient said that all his complaints had been burned away, and that he was quite well. Nothing remarkable occurred after this, and he quitted the hospital cured in the usual time.

Our author next proceeds to declare his opinion that the disease is a local one, the development of which it is possible to prevent, taking care to expel the virus; for, when *re-absorption* has once taken place, no means we are acquainted with can prevent a fatal result: from whence he concludes, that the known symptoms of hydrophobia, all of them highly nervous, are the effects of the re-absorption of the virus, which then produces universal disturbance. The virus first exercises its action upon the nerves of the sublingual and submaxillary glands, the nerves of the fifth pair, and those that go to the tongue.

Our author next enters into some physiological discussions, supported by a most extraordinary detail relative to the puppies of a bitch becoming hydrophobic, whilst the mother, who had been bitten, escaped; and which we cannot venture to relate, since it touches so closely upon the marvellous. From this and other circumstances, he concludes by declaring his belief in the existence of two species of hydrophobia,—one the slow species, in which the venom is either communicated in smaller quantities or in parts less vital, and here there are no pustules formed; the other appearing in a few days, and attended with pustules, which may be called the acute hydrophobia. We do not think it necessary to give our author's explanation of the causes of this difference in the progress of the symptoms.

In the last chapter, Dr. Marochetti makes some general observations on the appearance of the dog when affected with hydrophobia, in order that not only physicians, but all those who may have occasion to witness these accidents, may be competent to judge whether it is necessary to adopt precautionary measures, or to suffer the wounds to be healed by ordinary means, or left entirely to take a natural course. Therefore, to obviate any unfortunate mistake that might occur relative to the condition of the suspected animal, the following circumstances demand our attention:—1st, Its gait; 2d, its posture when reposing; 3d, its look; 4th, the appearance of the body; 5th, the change observable in its usual habits; 6th, the inversion of its instincts; 7th, its horror both of food and drink. The gait of a hydrophobic dog is unequal; he does not walk straight, but goes to the left or right, tottering, and often turns back again, so as not to appear to have any object in view; sometimes he runs, then stops suddenly, and falls down as if oppressed by a great weight. His posture is not the same as in a state of health; that is, he does not sit, but rests, as it were, upon his four paws, the front ones spread out to a great distance from each other. His aspect is fixed and melancholy, and, amidst the redness and fire of the eye, tears in abundance are to be seen; his body is extenuated, the tail hid between his legs; his limbs are half bent; the head held low, the ears hanging down, and the tongue and lips covered with foam. His habits are changed; for he avoids the house, as well as those persons to whom he was attached; he does not reply to his master's call, but appears even afraid of him; in general, he avoids both the society of man and his own species. His instincts are inverted; for he no longer serves as a guard; he ceases to bark, he knows no one, and frequents solitary places. Finally, neither food nor drink have any attractions for him; he sometimes, indeed, appears to wish for nourishment, but has scarcely taken a few mouthsful, when he is attacked by violent convulsions. The mere sight of a liquid, or even throwing it upon the body, produces inexpressible agonies, although it is evident he suffers greatly from thirst.

Cats present the same symptoms as dogs, but they are more dangerous, because they take most extraordinary leaps, particularly attacking human species. The rabid horse takes astonishing leaps, and runs without an object; when he meets with man, he bites, and tries to tread him under his feet. The milk of mares so affected will give the disease. The development of hydrophobia in consequence of the bite of a rabid wolf, is generally produced in a shorter time than by the bite of a dog, probably on account of the greater severity of the wounds. Our author knows no particular symptoms attending hydrophobia in that animal.

In this article, we have done little more than translate the observations of M. Marochetti: we would not be understood as agreeing with all the opinions of that gentleman.

